# A New Breed of Model: Estimating the Impact of Climate Change on Malaria Transmission

**DOI:** 10.1289/ehp.121-A310

**Published:** 2013-10-01

**Authors:** Claudia M. Caruana

**Affiliations:** **Claudia M. Caruana** is a New York–based health and science reporter.

Malaria is a serious global health issue, resulting in an estimated 219 million cases and 660,000 deaths in 2010, many of them in Africa.^1^ Malaria transmission is tied closely to environmental variables such as rainfall and temperature—even when there’s plenty of rainfall to produce breeding pools for the *Anopheles* mosquitoes that spread malaria, hot temperatures can hamper mosquito development.^2^ Some early projections predicted that climate change would cause an increase in malaria cases,^3^ but more recent reports suggest it’s more likely that cases will shift in their distribution rather than rise overall.^4^ In this issue of *EHP* investigators at the Massachusetts Institute of Technology (MIT) report their projections, using a new modeling tool, that there probably will not be a significant increase in malaria prevalence in West Africa, even during a worst-case scenario of increased rainfall in the region.^5^

The authors used the Hydrology, Entomology, and Malaria Transmission Simulator (HYDREMATS) to estimate the impact of climate change on malaria transmission in West Africa. HYDREMATS is a combined hydrology and entomology model of malaria transmission developed at MIT by coauthor Elfatih A.B. Eltahir, a professor in the Department of Civil and Environmental Engineering, and former graduate student Arne Bomblies, now an assistant professor at the University of Vermont. The model uses high-resolution data on environmental variables including rainfall, temperature, topography, and soil conditions to model ephemeral breeding pools that form during intense rains. The model also tracks the simulated behavior of individual mosquitoes as they interact with their environment.

The researchers used current climate data to model vectorial capacity, a measure of how efficiently mosquitoes spread malaria. They then looked at climate predictions for the time period 2080–2099 and determined which combination of temperature and rainfall changes corresponded to best- and worst-case scenarios in terms of malaria transmission. They conducted simulations using the best- and worst-case climate projections to predict vectorial capacity under each new scenario. The model did not include changes in malaria transmission due to interventions such as spraying, mosquito netting, and preventive medications.

**Figure 1:**
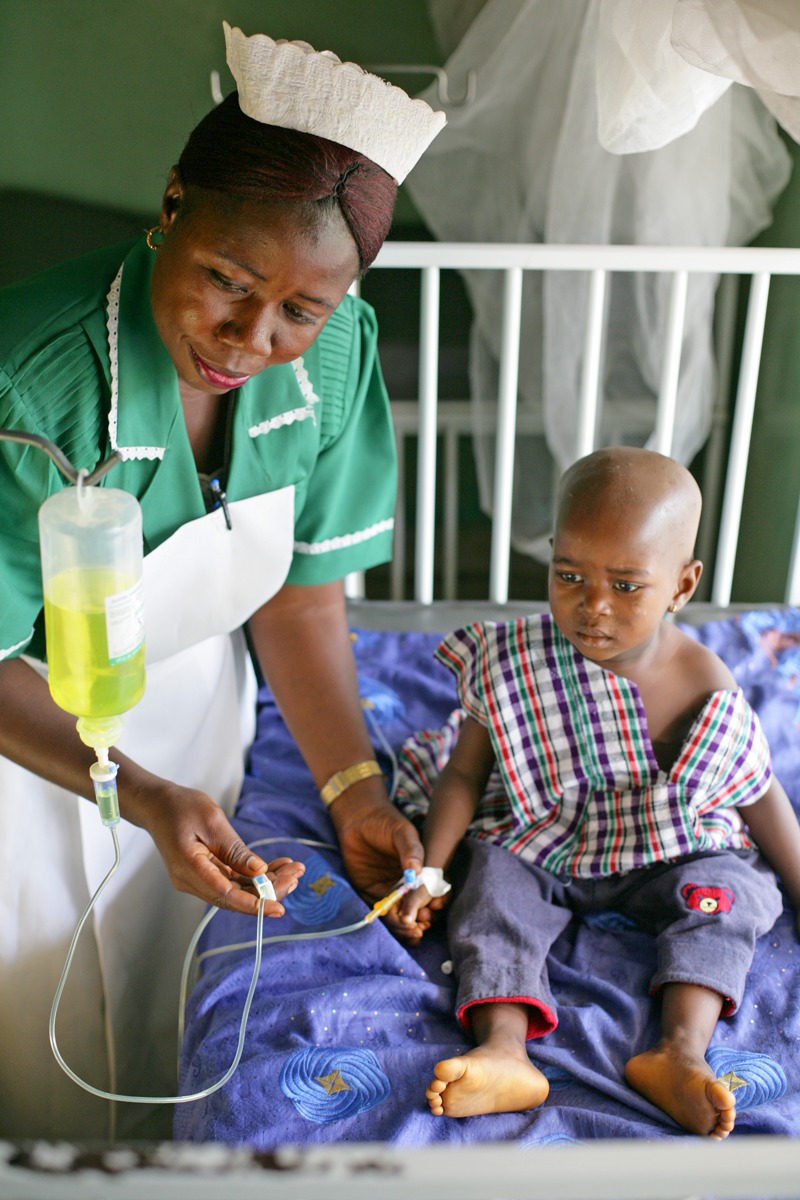
A child with malaria receives care in Sierra Leone. This country lies in a part of West Africa that is already saturated with malaria, and prevalence is not projected to increase with climate change. © Giacomo Pirozzi/Panos Pictures

**Figure 1:**
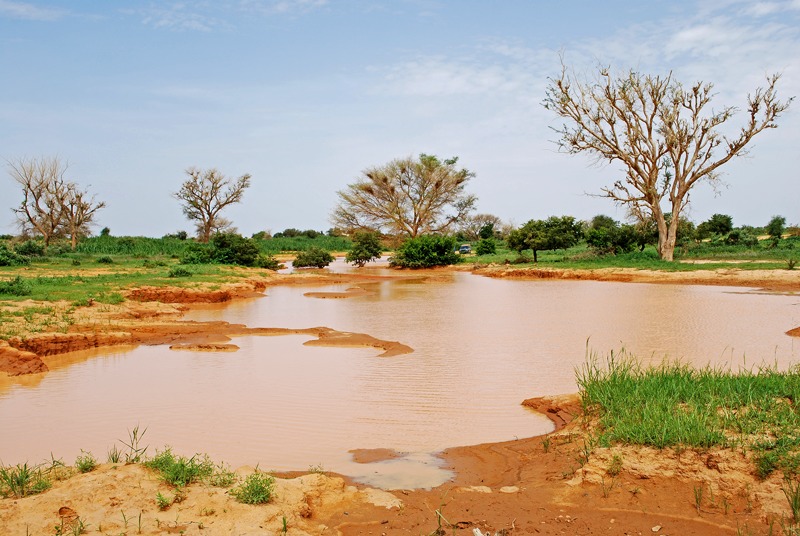
An ephemeral pool in Niger provides a perfect breeding site for Anopheles mosquitoes. This and other northern parts of West Africa could become too hot to sustain malaria. © Teresa Yamana/MIT

The northernmost areas studied are currently too dry and warm for effective malaria transmission. According to the model, they could become more suitable only if the climate becomes substantially wetter, but even then high temperatures likely would prohibit sustained transmission. The middle areas are expected to see a decrease in suitability for malaria transmission even under the wettest predictions of future climate. Southern areas could become even more suitable for transmission, but the persistent prevalence of malaria in these areas means a rise in cases is unlikely unless many people immigrate. Therefore, the investigators conclude, it appears unlikely, on the basis of this model, that climate change will increase malaria transmission in West Africa.^5^

“The main advantage of our malaria transmission model is that it provides a more detailed and direct relationship among environmental variables and malaria transmission than previous models,” says coauthor Teresa K. Yamana, a PhD student. “This is especially true for rainfall, because the timing of rain is just as important as the amount of rain. For example, more puddles form if there’s a big storm compared to if the same amount of rain falls over the course of several days.”

Another strength of the study is its consideration of a wide range of climate predictions. Yamana explains that climate impact studies may be based on the climate predictions of a single model without knowing whether that model accurately represents the region of interest. Others average the predictions made by multiple models, but this is not a good strategy in the case of West Africa: “Half of the predictions say the climate will be wetter, half say it will be drier,^6^” she says, “so the average is something close to no change in rainfall—this could end up being very far from the truth.”

Jonathan Patz, director of the Global Health Institute at the University of Wisconsin–Madison, is impressed by the researchers’ modeling because it “included a range of best- and worst-case scenarios to avoid bias. They also considered both temperature and rainfall, essential for malaria estimates.” He says, “Their findings are consistent with expectations that temperature projections alone explain only a part of malaria risk, and disease risk will considerably depend on rainfall and other environmental factors, particularly hydrological dynamics that vary by location.”
